# Physical Activity and Community Engagement (PACE) to facilitate community reintegration among returning veterans: Study protocol for a randomized controlled trial

**DOI:** 10.1016/j.conctc.2018.07.005

**Published:** 2018-07-25

**Authors:** Scarlett O. Baird, Christopher Metts, Haley E. Conroy, David Rosenfield, Jasper A.J. Smits

**Affiliations:** a Department of Psychology, Institute for Mental Health Research, University of Texas at Austin, Austin, TX, USA; b Department of Pathology and Laboratory Medicine, Medical University of South Carolina, Charleston, SC, USA; c Department of Psychology, Southern Methodist University, Dallas, TX, USA

## Abstract

There is a surprising lack of disseminable, community-based interventions for veterans experiencing difficulties during the reintegration process from military to civilian life. Physical Activity and Community Engagement (PACE) is a program which combines routine vigorous-intensity exercise with community engagement. The program builds on emergent evidence supporting the benefits of routine vigorous-intensity exercise among and establishing social connection. Using a randomized controlled trial (N = 60), we will obtain feasibility data and initial effect sizes for the early effects of PACE on reintegration difficulties.

## 1 Introduction

Since September 11th, 2.77 million service members have served on 5.4 million deployments [26]. While the majority of veterans reintegrate successfully following deployment, a sizable minority return and face difficulties transitioning into civilian life. Specifically, as many as 44% of post 9/11 veterans report that the readjustment to civilian life was difficult [22]. Recent estimates have shown that at least 25% of combat veterans who used VA medical services endorse “some” to “extreme” difficulty adjusting to civilian life (Sayer et al., 2010). Stigma has been documented as a barrier to seeking help among Operation Enduring Freedom (OEF) and Operation Iraqi Freedom (OIF) veterans [9,17]. Thus, developing accessible programs focused on facilitating reintegration is imperative [19].

The U.S. Department of Defense (DoD) does not provide a formal definition of successful reintegration. However, they have worked to address the issue through the development of mandatory transition assistance programs within each service branch (for review see Ref. [27]. These programs are often implemented within the context of a multitude of other administrative procedures that a service members must complete upon the return home. Accordingly, while a crucial first step, these existing programs leave room for additional intervention following discharge.

Physical Activity and Community Engagement (PACE) is a program that centers on routine vigorous-intensity exercise and social connection. Keeping dissemination in mind, PACE was designed such that it can easily be “prescribed” to augment existing transition assistance programs. Support for helping veterans adopt a vigorous-intensity exercise routine comes from numerous trials indicating that engaging in exercise improves mood and reduce symptoms of depression and anxiety (for review see Ref. [16], which in turn facilitates community reintegration. Indeed, compared to other levels of exercise intensity, vigorous-intensity exercise has been associated with increases in well-being [5], reductions in anxiety and depression [1,4,11] and improvements in quality of life [14]. Importantly, high intensity exercise is a leading trend in the fitness industry [25] and has gained increasing popularity particularly within military units. For example, CrossFit, a high intensity exercise program, now has affiliates on a large number of military installations across the country [8].

Founded in 2010, Team Red White and Blue (RWB) is a non-profit organization that aims to enrich the lives of veterans by enhancing their connection to their community. The organization offers recurrent weekly activities with the aim of encouraging both veterans and civilians to interact and support each other in the local community. With over 200 chapters across the United States, RWB has the potential to reach most veterans as they transition into civilian life. The chapters target social connection by organizing activities, including exercise groups (e.g., running, yoga, biking), social activities (e.g., coffee meetups, trivia nights, dinners), and community service events. Initial survey findings support the mission by showing that involvement in Team RWB (1) increases connectivity; (2) helps bridge the civilian/military divide; and (3) improves well-being and life satisfaction (“ [23]: Enriching the Lives of America's Veterans,” 2014). Although making available the opportunity to participate in exercise, RWB does not provide guidelines for dosing nor are there expectations for meeting specific exercise goals. Hence, although it has the potential to engage the social connection target by offering multiple opportunities to participate in various activities, it is plausible that RWB would offer better outcomes for veterans experiencing reintegration difficulties if it was combined with brief exercise counseling and monitoring aimed at helping them adopt a vigorous-intensity exercise routine. The PACE program provides recently discharged veterans experiencing difficulties with reintegration a referral to RWB as well as exercise counseling and monitoring.

The study aims to provide a pilot test of the acceptability and effectiveness of PACE in a sample of recently discharged veterans. To our knowledge, this is the first study that examines a multi-component intervention for facilitating community reintegration in veterans during this critical period of the deployment cycle.

## 2 Methods

### 2.1 Study design

Sixty veterans discharged from the U.S. military will be randomly assigned to: 1) PACE, which includes vigorous-intensity exercise counseling and monitoring in addition to participation in a community-based program (Team RWB) or (2) 8 participation in Team RWB alone or (3) waitlist. The project will be a collaboration between the University of Texas at Austin (UT) and Team RWB. UT Austin will conduct all research activities and refer participants to Team RWB activities. The primary outcome – community reintegration difficulties - will be assessed at baseline, biweekly during the intervention period, and after 8 weeks of the intervention. PACE and Team RWB aim to aid the development of long-term habits and thus are not time-limited, Hence, the current study will report on the early effects of these programs – i.e., bi-weekly during the first 8 weeks and 1-week following 8 weeks of participation.

### 2.2 Specific aims

1. To assess, in a randomized clinical trial, the feasibility and acceptability of the PACE program. We will examine participant attendance to prescribed activities as well as participant satisfaction with the program using an acceptability interview following study completion.

2. To evaluate the effectiveness of the PACE program on the level of reintegration difficulties on the following primary outcome measure: Military to Civilian Questionnaire (M2C-Q; [20].

### 2.3 Participants

Participants will be 60 veterans who have completed the discharge process within the last 5 years. Participants will be veterans of the OEF/OIF/Operation New Dawn (OND) conflicts. The participants will have to endorse at least moderate difficulty with community reintegration. Moderate difficulty will be defined using a two-pronged approach: participants must 1) score ≥1.5 on the M2C-Q, and 2) also endorse at least a “2” (i.e., “some difficulty”) on item 14 (feeling as if one belongs in civilian society). Other inclusion/exclusion criteria include: 1) Participants must have access to an Apple iPhone. This criterion is required due to the need to use the Apple Watch and the study App (Status/Post). Both components are required for data collection, 2) Participants must have participated in less than 75 min of vigorous-intensity exercise per week over the two weeks prior to screening, 3) Participants must be willing and able to comply with an 8-week protocol, 4) Participants must have sufficient command of the English language to use the study's App and to fill out the study questionnaires, and 5) Participants must not have a condition or injury which would prevent vigorous-intensity exercise. Participants will complete the Physical Activity Readiness Questionnaire (PAR-Q) as part of the screening procedure in order to check for any condition/injury which would render exercise harmful. Each participant will also have undergone a routine physical with medical staff prior to discharge and thus has knowledge of a condition or injury. If such a risk is present, the participant will be excluded from the study. If the participant is unsure of potential risk, medical clearance from a physician will be required prior to enrollment.

### 2.4 Procedures

The Institutional Review Board of the University of Texas at Austin approved the study. The study is currently in the recruitment phase.

### 2.5 Recruitment

Participants will be recruited from multiple cities nationwide. The study will use brief text announcements on various online platforms (e.g., Facebook, Reddit, Craigslist). Research staff will also attend various veteran community events in order to promote the study and highlight potential benefits.

### 2.6 Screening

Individuals interested in participating in the study will be directed via various recruitment strategies to an Internet prescreen using Research Electronic Data Capture (REDCap). Each participant will be required to read through an informed consent form and either agree/not agree to provide his/her information prior to beginning the survey. This online prescreen will assess basic eligibility criteria, and individuals who appear potentially eligible will be further assessed via a telephone prescreen. The prescreen procedure is the first point of contact for participants, and it will allow us to assess the potential participant's willingness and ability to commit to the intervention as well as ensure the assessment of inclusion/exclusion criteria. If eligible following the phone screen, participants will be invited to schedule a baseline assessment.

### 2.7 Baseline/enrollment

For individuals able to commute to the study site, the visit will take place on campus at UT Austin. For those who are unable to come to the study site, the visit can take place via videoconference (e.g., Facetime). Prior to any assessment, participants will receive an online informed consent form explaining the details of the study, potential benefits and risks of participation, and the procedures they will undergo if they choose to participate. After reading the informed consent, research staff will discuss any issues with the potential participant and will answer any questions he or she may have about the study and participation. If the individual chooses to sign the informed consent, he/she will begin the baseline visit.

During this visit, research staff will first administer the baseline assessment (see Assessment Schedule). Upon completion of the measures, the researcher will then inform the veteran of randomization assignment, provide the Apple Watch and instructions, and provide compensation ($20). If the participant is not in Austin or the Fort Hood/Killeen area, the watch will be shipped and the session will take place via video conference once the watch is received.

Each participant will be provided with an Apple Watch to wear for the duration of the study. Participants will be asked to sign a contract indicating that they are aware that the equipment belongs to UT. However, should the participant complete the follow up and at least two of the additional biweekly surveys, he/she will be given the watch. Research staff will use the Apple Watch to monitor the participant's activity level. In addition, each participant will be sent a brief automated survey via REDCap each day which will take no more than 1–2 min to complete using the Status/Post app installed on their Apple iPhone for the purpose of the study. The survey will ask whether the participant has completed exercise that day, and if so, it asks to enter the type of exercise, number of minutes exercised, average heartrate, and perceived exertion.

### 2.8 Intervention modules

#### 2.8.1 Team RWB

Participants assigned to the Team RWB arm will be prescribed 1 Team RWB event per week for the 8-week duration of the study. Participants will be guided through the process of joining Team RWB. Research staff will have the participant “Join the Team” on the organization's site: https://www.teamrwb.org/join/. Research staff will connect the participant via email to the correct chapter representative based on which chapter the participant expresses interest in. In this way, research staff can ensure that the participant is connected with the organization in a timely manner. Participants will receive a monthly calendar of Team RWB events upon assignment. They will be asked to attend at least one Team RWB event. This could be a weekly running group, social event, or community service project, among other activities. We did not discourage the use of exercise in this arm in order to optimize the ecological validity of the program.

Participants assigned to this condition will also enter whether or not they attended a Team RWB event on the daily automated REDCap surveys. Participants will be asked to not initiate an exercise program outside of the context of Team RWB for the duration of the study. However, if they do become involved in another fitness program, we will ask that they report this to study staff. The staff will save this information as a note to file and these data will be reported in the outcome report. In order to be able to evaluate the effects of Team RWB as it is administered, research staff will not provide any guidance to participants or participate in trouble-shooting non-adherence.

Study staff will schedule a total of four online surveys to be distributed via REDCap every two weeks. The surveys should take approximately 30 min to complete and participants will be compensated $20 per assessment.

#### 2.8.2 PACE

Participants assigned to the integrated arm will be prescribed the following for the course of 8 weeks: 1 session of exercise counseling, 3 weekly 25-minute sessions of vigorous-intensity aerobic exercise, and 1 weekly Team RWB event.

Research staff will complete a session of exercise counseling with the participant during the baseline visit and an exercise prescription form will be provided to the participant once completed. The overall aim of this session is to set a predetermined goal of activities and troubleshoot any barriers to activity. At this time, the participant will also indicate an exercise preference. Exercise will be limited to running, cycling, rowing, or elliptical workouts. By allowing participants to choose their preferred mode of exercise, we will be able to tailor the intervention to the individual and likely increase exercise adherence as a result.

Following the exercise counseling session, participants will be given a prescription form to remind them of their target heart rate during exercise sessions, exercise preference, and a schedule of planned exercise. The form will also list the goals that the participant identified during the exercise counseling session to serve as motivational reminders. Participants will receive a paper copy but will also be encouraged to take a picture on their personal device or input this information into their personal calendar. Research staff will then track the type of exercise the participant is completing (via the daily REDCap surveys) and send reminders if the participant fails to complete the weekly dose of exercise.

In accordance with the Centers for Disease Control and Prevention [CDC] guidelines, the exercise prescription will be three separate 25-minute sessions of vigorous-intensity aerobic exercise (running, cycling, rowing, or elliptical workouts) per week (for a minimum of 75 min). In order to ensure that participants are completing the prescribed dose of vigorous intensity aerobic exercise, study staff will monitor the weekly data from the participant's Apple Watch. Data collected will include the number of minutes exercised and the heartrate level (77–85% maximum heart rate). Any exercise session of 25 min at an average > 76% of maximum heart rate will be characterized as 1 completed session of vigorous intensity aerobic exercise. Furthermore, participants will be asked to enter the type of activity, number of minutes exercised, average heartrate, and perceived exertion into the exercise log on REDCap through the brief automated survey sent daily.

Participants will then complete the same procedure for joining Team RWB as participants in the Team RWB arm. Participant will be told that they can complete (part of) the weekly exercise prescription during Team RWB activities as long as the exercise activity meets the criterion of at least 25 min of vigorous-intensity exercise. They will enter attendance at Team RWB events on the daily REDCap surveys. Because PACE integrates routine vigorous-intensity exercise with team RWB participation, research staff will, in addition to exercise adherence, check participant's team RWB participation weekly. If the participant has missed the set goal of either exercise or a Team RWB event, staff will follow-up with a text message or email to remind the participant of his/her weekly goal. If needed, staff will schedule a phone call to enhance motivation and troubleshoot barriers. Staff will also provide feedback at the end of each week in the form of a text or email for participants, in this condition only. The content will inform the participant of progress toward his/her goal and provide encouragement as needed.

#### 2.8.3 Waitlist

Participants assigned to the waitlist arm will receive no study intervention and will only complete the scheduled assessments. Participants will be asked to not initiate a new exercise program or attend a Team RWB event for the duration of the study. However, if they do become involved in a fitness program or attend a chapter event, we will ask that they report this to study staff (i.e., on the automated surveys). The staff will save this information as a note to file and these data will be reported in the outcome report. After completion of the follow-up assessment, participants in the waitlist arm will be guided through the process of joining Team RWB.

#### 2.8.4 Follow-up

Participants will be asked to complete a final assessment on REDCap one week following study completion (i.e., week 9), which will serve as the major endpoint for initial efficacy. Participants who met the survey requirements (i.e., follow up and at least 2 of the biweekly assessments) will keep the Apple watch. Those who do not will be asked to return the Apple Watch within two weeks of the week 9 follow-up.

### 2.9 Randomization

The principal investigator will oversee the randomization. Randomization will be stratified by military rank (i.e., officer or enlisted status). Variable-sized permuted block randomization will be completed using Sealed Envelope to assign individuals to treatment condition (Sealed Envelope Ltd. 2016).

### 2.10 Assessments

All assessments will be delivered to participants using status/post, a mobile application developed for iOS devices, which participants download from the Apple App Store. Upon downloading the app, users enter their assigned username and password to gain access to our study in the app. Upon app initialization, the participant is prompted to give permission for the app to send notifications and collect heart rate data. After permission is given, the app will notify the participant of surveys needing completion at scheduled times and begin collecting heart rate data that the participant's Apple Watch has deposited in Apple's Health app.

All iOS apps are “sandboxed,” meaning the other apps cannot gather data collected by the study app, and likewise, the study app cannot gather data from other apps. All data on the iPhone is encrypted, and collected data is not available to users of the iPhone. Collected data is sent to REDCap using the provided API, and this data transmission is encrypted with SSL. UT's REDCap installation provides HIPAA compliance for data stored in REDCap. Additional biweekly assessments will also be sent via the application. If the participant does not complete the necessary number of assessments, he/she will be instructed to reset the watch and restore to factory settings upon returning the equipment. In this way, researchers will not have access to any of the participant's data following the study.

The Apple Watch will be shipped to participants who are unable to come to the lab for the baseline assessment, and a call will be scheduled to ensure that they are given the same set up instructions. Participants will then receive information regarding study assignment and will be asked to download the study application (Status/Post) in order to be able to complete assessments over the course of the study.

### 2.11 Screening and baseline

Demographics. Participants will be asked to provide standard demographic information (age, gender, race/ethnicity, level of education, marital status). We will also assess for participants' rank at the time of discharge, branch of service, discharge date, and number of deployments. Demographics will be assessed during the prescreen and at the baseline assessment.

Physical Activity Readiness Questionnaire (PAR-Q; [24]. This measure is used to assess the safety and potential risk of exercise. It looks at the individual's history, current symptoms, and risk factors. This measure has been adapted and is part of the American College of Sports Medicine (ACSM)'s guidelines for exercise testing.

Military to Civilian Questionnaire (M2C-Q; [20]. The measure is a 16-item self-report measure of postdeployment community reintegration difficulty. Participants must endorse at least moderate reintegration difficulties. See below for details of this measure.

### 2.12 Primary outcome

Military to Civilian Questionnaire (M2C-Q). The primary outcome measure will be the level of reintegration difficulties (as evidenced on the Military to Civilian Questionnaire [M2C-Q]). The measure is a 16-item self-report measure of post-deployment community reintegration difficulty. The measure is rated on a 5-point Likert scale with the following responses: “0 = No difficulty”, “1 = A little difficulty”, “2 = Some difficulty”, “3 = A lot of difficulty”, and “4 = Extreme difficulty”. Scores on each item are then averaged for a total score. We chose a cut off of 1.5 in combination with a “2” on item 14 as a measure of moderate difficulty in adjusting to civilian life. Item 14 asks the main target that we want to address (i.e., belonging in civilian society; [20]. The measure demonstrates high internal consistency (Cronbach's α = 0.95).

### 2.13 Secondary outcomes

The Alcohol Use Disorders Identification Test (AUDIT). The AUDIT-C is a 3-item alcohol screen that can help identify persons who are hazardous drinkers or have active alcohol use disorders (including alcohol abuse or dependence; [3]. In men, a score of 4 or more is considered positive, optimal for identifying hazardous drinking or active alcohol use disorders. In women, a score of 3 or more is considered positive (same as above). However, when the points are all from Question #1 alone (#2 & #3 are zero), it can be assumed that the individual is drinking below recommended limits and it is suggested that the provider review the individual's alcohol intake over the past few months to confirm accuracy. Generally, the higher the score, the more likely it is that the individual's drinking is affecting his or her safety. This measure will be administered at baseline and at the final follow-up assessment.

Primary Care PSTD Screen for DSM-5 (PC-PTSD-5). This 5-item measure was designed for use in primary care and medical settings [18]. The measure is used to screen for probable PTSD. The results typically suggest a probably diagnosis of PTSD if at least 3 of the 5 items are answered “yes.” This measure will be administered at baseline and at the final follow-up assessment.

The PTSD Checklist for DSM-5 (PCL-5). The PCL-5 is a 20-item self-report measure that assesses the symptoms of PTSD [2]. The measure can be used to screen individuals for the disorder, make a provisional diagnosis, or to monitor symptom change. For the purposes of the study, the measure will be used to monitor symptom severity and change for those with a probable diagnosis of PTSD (as measured by the PC-PTSD-5). It will be administered for individuals who score a 3 or above on the PC-PTSD-5 at the baseline and the final follow-up assessments.

The Patient Health Questionnaire-9 (PHQ-9). The PHQ-9 is a 9-item measure designed to screen for and monitor the severity of depressive symptoms. The measure has demonstrated diagnostic validity and how shown to be both sensitive and specific to major depressive symptoms. Scores of 5, 10, 15, and 20 are indicative of mild, moderate, moderately severe, and severe depression [12]. The measure will be included in each assessment.

Meaning in Life Questionnaire (MLQ). This is a 10-item measure designed to measure two dimensions of meaning in life: 1) presence of meaning (how much participants feel that their lives have meaning) and 2) search for meaning (how much participants strive to find meaning in their lives). The MLQ has demonstrated good internal consistency with coefficient alphas ranging in low to high 0.80s for the Presence subscale and mid 0.80s to low 0.90s for the Search subscale [21]. The measure will be included in each assessment.

Satisfaction with Life Scale (SWLS). This is a 5-item instrument designed to measure global cognitive judgments of satisfaction with one's life [6]. The SWLS is a widely-used measure of well-being and has demonstrated validity across a number of studies (for review, see Ref. [15]. The measure will be included in each assessment.

Brief Sensation Seeking Scale (BSSS). The BSSS is an 8-item instrument designed to measure sensation seeking, a dispositional trait found to be a risk factor for various problematic behaviors. The scores have been shown to be reliable and predictably associated with a number of risk and protective factors [10]. Previous work suggests that sensation seeking plays an important role in the long-term adjustment period following war [13]. The measure will be included in each assessment.

Team RWB Enriched Life Scale (ELS). The Team RWB ELS is a 48-item instrument designed to measure an “enriched” life defined as positive health, genuine relationships, and sense of individual and shared purpose. Items were derived based upon a review of the literature and Team Red, White & Blue's annual member survey of 16,370 veterans, active duty service members, and civilians in 2014, 2015, 2016. Validity and reliability will be established in veteran service member, civilian, and law enforcement populations in early 2017. The measure will be included in each assessment.

### 2.14 Vigorous-intensity exercise adherence prescription integrity

Vigorous Intensity Exercise Adherence. Participant adherence in the integrated study arm will be assessed with the daily REDCap surveys and with the Apple Watch heartrate data. Participants will be asked to enter the type of physical activity and number of minutes exercised, average heartrate, and perceived exertion into the daily REDCap exercise adherence survey. For the individuals in the PACE condition who fail to complete the prescribed weekly dose, brief reminder calls will be made by the research staff. The calls are designed to: (1) foster motivation and impetus for behavioral change, and (2) troubleshoot potential barriers to physical activity.

Heart rate will be monitored with the Apple Watch. Participants will be asked to wear the Apple Watch every day as well as for each session of exercise. The watch will monitor the level of heart rate and provide the average heart rate for the session. Participants will be asked to enter this information on the daily REDCap survey. In addition, Status/Post will pull the heart rate data from HealthKit on the Apple Watch and mobile device, allowing us to verify transcribed data. A participant will meet the weekly prescription if they have evidenced at least 75 min of at least 76% of maximum heart rate.

### 2.15 Team RWB prescription integrity

Participant adherence will be assessed using the daily REDCap adherence surveys. For the individuals in the PACE group who failed to attend a weekly event, brief reminder calls will be made by the research staff. The calls are designed to: (1) foster motivation and impetus for behavioral change, and (2) troubleshoot potential barriers to community engagement. Researchers will use the call as an opportunity to encourage the participant to attend at least one event the following week. Research staff will not schedule reminder calls for those in the Team RWB alone arm in order to ensure that this group is a pure test of the current organization and procedures.

### 2.16 Acceptability

Acceptability Interview. This measure will assess participants' perceptions of the PACE program in terms of likelihood of future engagement, program likeability, and perceived benefits of the intervention (e.g., “Some people feel like other things going on in their lives have impacted their experience in this program. What, if any, things in your life outside of the program impacted your experience?” or “What did you like or find helpful about the PACE program; what benefits have you noticed?”) The measure will be administered at follow-up online via REDCap.

## 3 Data analysis

As the project is designed to be a proof-of-concept, the primary aim is to gather information about acceptability as well as initial effect sizes of the PACE program. In this way, the data can help to guide treatment development and inform future efficacy studies. In order to examine the acceptability of the PACE program, we will report on intervention adherence (e.g., vigorous-intensity exercise adherence, Team RWB attendance) as well as items from the acceptability interview.

We will initially examine baseline differences between groups on various demographic and psychological variables (e.g., military rank, gender, reintegration difficulties) to ensure that there are no prior differences between groups. Any variables on which the groups differ will be used as covariates in subsequent analyses. Effect sizes of PACE relative to WL or RWB only, respectively, will be estimated using [7] adaptation of Cohen's *d* to mixed effects models. Effect size analyses will be complemented by traditional significance testing employing repeated ANCOVA using mixed effects models with the M2C-Q as the dependent variable, time as the within-subjects variable and intervention group (Team RWB + Vigorous-Intensity Exercise, Team RWB, WL) as the between-subjects variable.

### 3.1 Power analyses

The primary objective during this developmental phase is to estimate the effect size of the PACE program. Thus, we recognize that the study is not powered to detect small statistical differences between the three groups. A post hoc power analysis (power = 0.8, α = 0.05) indicated that the final model, with the sample size of 60 participants, allowed us to detect a significant medium effect size of *f*^*2*^ = 0.25 for the between by within subject interaction.

## 4 Discussion

While there is a sizable literature pointing to the difficulties associated with reintegration for returning service memberss [9]; Sayer et al., 2010; [22], there is a large gap in the research of evidence-based community programs to reduce such difficulties. Veterans experience difficulties across a number of life domains (e.g., interpersonal relationships, feeling as if one belongs in civilian society, deriving a sense of purpose after the military). Many service memberss return to civilian society and either attempt to cope with their symptoms alone or are not seen until they reach a formal healthcare setting (e.g., the Veterans Affairs hospital). The PACE program seeks to engage veterans earlier on in the transition process. To this end, we may be able to catch veterans prior to serious difficulties arising or the development of a mental health disorder.

The study aims to address an important public health issue by evaluating the initial efficacy of this integrated prescription. The results should inform whether or not veterans would be willing to engage in a community-based program as well as whether or not such a program would be beneficial in reducing community reintegration difficulties. The expected findings should provide initial effect size data for PACE, and thus provide the necessary data for a large-scale follow-up trial.

## Financial disclosures

Jasper Smits receives funding from the NIH (R34DA034658, R34MH099318, R33MH109600, R34DA044431) and has received monetary compensation for his work as consultant to Microtransponder, Inc. and Aptinyx, Inc. He also received royalties from various book publishers. Christopher Metts, MD is the developer of the status/post mobile application used in this study. Development was conducted in his role as President of Infinite Arms, LLC, which maintains ownership of the Intellectual Property associated with this application.

## Appendix A Supplementary data

Data Profile Data Profile

## References

[1] Balchin R., Linde J., Blackhurst D., Rauch L.H., Schönbächler G. (2016). Sweating away depression? The impact of intensive exercise on depression. J. Affect. Disord..

[2] Blevins C.A., Weathers F.W., Davis M.T., Witte T.K., Domino J.L. (2015). The posttraumatic stress disorder checklist for DSM‐5 (PCL‐5): development and initial psychometric evaluation. J. Trauma Stress.

[3] Bush K., Kivlahan D.R., McDonell M.B., Fihn S.D., Bradley K.A. (1998). The AUDIT alcohol consumption questions (AUDIT-C): an effective brief screening test for problem drinking. Arch. Intern. Med..

[4] Cox R.H., Thomas T.R., Hinton P.S., Donahue O.M. (2004). Effects of acute 60 and 80% VO_2_ bouts of aerobic exercise on state anxiety of women of different age groups across time. Res. Q. Exerc. Sport.

[5] Cox R.H., Thomas T.R., Hinton P.S., Donahue O.M. (2006). Effects of acute bouts of aerobic exercise of varied intensity on subjective mood experiences in women of different age groups across time. J. Sport Behav..

[6] Diener E., Emmons R.A., Larsen R.J., Griffin S. (1985). The satisfaction with life scale. J. Pers. Assess..

[7] Feingold A. (2013). A regression framework for effect size assessments in longitudinal modeling of group differences. Rev. Gen. Psychol..

[8] Haddock C.K., Poston W.S.C., Heinrich K.M., Jahnke S.A., Jitnarin N. (2016). The benefits of high- intensity functional training fitness programs for military personnel. Mil. Med..

[9] Hoge C.W., Castro C.A., Messer S.C., McGurk D., Cotting D.I., Koffman R.L. (2004). Combat duty in Iraq and Afganistan, mental health problems, and barriers to care. N. Engl. J. Med..

[10] Hoyle R.H., Stephenson M.T., Palmgreen P., Lorch E.P., Donohew R.L. (2002). Reliability and validity of a brief measure of sensation seeking. Pers. Indiv. Differ..

[11] Katula J.A., Blissmer B.J., McAuley E. (1999). Exercise intensity and self- efficacy effects on anxiety reduction in healthy, older adults. J. Behav. Med..

[12] Kroenke K., Spitzer R.L., Williams J.B.W. (2001). The PHQ-9: validity of a brief depression severity measure. J. Gen. Intern. Med..

[13] Neria Y., Solomon Z., Ginzburg K., Dekel R. (2000). Sensation seeking, wartime performance, and long- term adjustment among Israeli war veterans. Pers. Indiv. Differ..

[14] Ostman C., Jewiss D., Smart N.A. (2017). The effect of exercise training intensity on quality of life in heart failure patients: a systematic review and meta-analysis. Cardiology.

[15] Pavot W., Diener E. (1993). Review of the satisfaction with life scale. Psychol. Assess..

[16] Penedo F.J., Dahn J.R. (2005). Exercise and well-being: a review of mental and physical health benefits associated with physical activity. Curr. Opin. Psychiatr..

[17] Pietrzak R.H., Johnson D.C., Goldstein M.B., Malley J.C., Southwick S.M. (2009). Percieved stigma and barriers to mental health care utilitzation among OEF- OIF veterans. Psychiatr. Serv..

[18] Prins A., Bovin M.J., Smolenski D.J., Marx B.P., Kimerling R., Jenkins-Guarnieri M.A., Tiet Q.Q. (2016). The primary care PTSD screen for DSM-5 (PC-PTSD-5): development and evaluation within a veteran primary care sample. J. Gen. Intern. Med..

[19] Sayer N.A., Noorbaloochi S., Frazier P.A., Pennebaker J.W., Orazem R.J., Schnurr P.P., Murdoch M., Carlson K.F., Gravely K.F., Litz B.T. (2015). Randomized controlled trial of online expressive writing to address readjustment difficulties among U.S. Afganistan and Iraq war veterans. J. Trauma Stress.

[20] Sayer N.A., Frazier P., Orazem R.J., Murdoch M., Gravely A., Carlson K.F., Hintz S., Noorbaloochi S. (2011). Military to civilian questionnaire: a measure of postdeployment community reintegration difficulty among veterans using department of veterans Affairs medical care. J. Trauma Stress.

[21] Steger M.F., Frazier P., Oishi S., Kaler M. (2006). The Meaning in Life Questionnaire: assessing the presence of and search for meaning in life. J. Counsel. Psychol..

[22] Taylor P., Morin R., Parker K., Cohn D.V., Funk C., Mokrzycki M. (2011). War and Sacrifice in the Post-9/11 Era.

[23] (2014, September 8). Team Red White and Blue Study: Enriching the Lives of America's Veterans.

[24] Thomas S., Reading J., Shephard R.J. (1992). Revision of the physical activity readiness questionnaire (PAR-Q). Can. J. Sport Sci..

[25] Thompson W.R. (2013). Now trending: worldwide survey of fitness trends for 2014. ACSM's Health & Fit. J..

[26] Wenger J.W., O'Connell C., Cottrell L. (2018). Examination of Recent Deployment Experience across the Services and Components.

[27] Yosick T., Bates M., Moore M., Crowe C., Phillips J., Davison J. (2012). A Review of post-deployment Reintegration: Evidence, Challenges, and Strategies for Program Development.

